# Tea Grape Reduces Abdominal Aortic Occlusion-Induced Lung Injury

**DOI:** 10.21470/1678-9741-2019-0392

**Published:** 2020

**Authors:** Doğuş Hemşinli, Saban Ergene, Sedat Ozan Karakişi, Tolga Mercantepe, Levent Tumkaya, Adnan Yilmaz, Kerimali Akyilzdiz

**Affiliations:** 1 Department of Cardiovascular Surgery, Recep Tayyip Erdogan University, Faculty of Medicine, Rize, Turkey.; 2 Department of Histology and Embryology, Recep Tayyip Erdogan University, Faculty of Medicine, Rize, Turkey.; 3 Department of Medical Biochemistry, Recep Tayyip Erdogan University, Faculty of Medicine, Rize, Turkey.; 4 Department of Medical Services and Techniques, Health Care Services Vocational School, Recep Tayyip Erdogan University, Rize, Turkey.

**Keywords:** Lung Injury, Alveolar Epithelial Cells, Glutathione, Glycerol, Aorta, Abdominal, Lung, Reperfusion, Ischemia, Oxidative Stress, Apoptosis, Edema, Rats

## Abstract

**Introduction:**

Ischemia-associated mortality caused by aortic cross-clamps, as in ruptured abdominal aorta aneurysm surgeries, and reperfusion following their removal represent some of the main emergency conditions in cardiovascular surgery. The purpose of our study was to examine the potential protective effect of tea grape against aortic occlusion-induced lung injury using biochemical, histopathological, immunohistochemical, and quantitative analyses.

**Methods:**

Thirty-two male Sprague-Dawley rats were randomly assigned into four groups: control (healthy), glycerol + ischemia/reperfusion (I/R) (sham), I/R, and I/R + tea grape.

**Results:**

Following aortic occlusion, we observed apoptotic pneumocytes, thickening in the alveolar wall, edematous areas in interstitial regions, and vascular congestion. We also observed an increase in pulmonary malondialdehyde (MDA) levels and decrease in pulmonary glutathione (GSH). However, tea grape reduced apoptotic pneumocytes, edema, vascular congestion, and MDA levels, while increased GSH levels in lung tissue.

**Conclusion:**

Our findings suggest that tea grape is effective against aortic occlusion-induced lung injury by reducing oxidative stress and apoptosis.

**Table:** 

Abbreviations, acronyms & symbols				
ALHDS	= Alveolar and Lung Histopathological Damage Score		IU	= International units
Gly	= Glycerol		LPO	= Lipid peroxidation
GSH	= Glutathione		MAP	= Mean arterial pressure
HCl	= Hydrochloride		MDA	= Malondialdehyde
i.p	= Intraperitoneal		RAAA	= Ruptured abdominal aorta aneurysm
I/R	= Ischemia/reperfusion		ROS	= Reactive oxygen species
IHC	= Immunohistochemical		TG	= Tea grape

## INTRODUCTION

Ischemia-induced mortality resulting from aortic cross-clamps used in ruptured abdominal aorta aneurysm (RAAA) surgeries and reperfusion resulting from their removal are among the main emergency conditions in cardiovascular surgery^[1,2]^. Although tissue damage associated with aortic occlusion has been examined in organs such as kidney, small intestine, and ovary, very few studies investigated its effects on the lung^[3-5]^. The mechanism underlying aortic occlusion-related tissue damage is not fully understood. Previous studies implicated reactive oxygen species (ROS)-mediated oxidative stress^[3,4,6,7]^. Several oxidant and antioxidant molecules and enzymes are involved in oxidative stress, the levels of which increase due to lipid peroxidation (LPO)^[8,9]^. This is also known to cause an increase in levels of malondialdehyde (MDA), the most important marker of LPO^[10]^. Several previous studies reported that glutathione (GSH) peroxidase plays an important role in reducing ROS^[11]^. Recent studies reported that ischemia/reperfusion (I/R) injury leads to caspase-dependent apoptosis associated with increased ROS, especially caspase-3, an irreversible terminal event in the activation of the apoptotic mechanism and one that plays a key role in ROS-related apoptosis^[12]^.

Due to its high anthocyanidin (flavonoid) content, tea grape (whortleberry, *Vaccinium myrtillus*) exhibits a range of biological effects including antioxidant, anti-inflammatory, anticarcinogenic, and antihyperlipidemic activities^[10,13]^. Tea grape has also been reported to eliminate ROS through decreased MDA and increased GSH levels^[14,15]^.

The purpose of this study was to investigate the potential protective effect of tea grape against aortic occlusioninduced lung injury using biochemical, histopathological, immunohistochemical (IHC), and quantitative analyses.

## METHODS

### Animals

Thirty-two Sprague Dawley rats weighing 232±23 g and aged 3-4 months were allocated into four groups of eight animals each: control, glycerol + I/R (sham), I/R, and I/R + tea grape. Rats were fed and housed at a room temperature of 22±1 °C and 55-60% humidity, in a 12-12-hour light/dark cycle in the experimental animals unit of the university. All animals received humane care according to the criteria outlined in the Guide for the Care and Use of Laboratory Animals prepared by the National Academy of Sciences and published by the National Institutes of Health. Approval for the study was granted by the Recep Tayyip Erdoğan University Animal Ethical Committee.

### Aortic Clamping Technique

In agreement with the RAAA model described in previous studies, the glycerol, I/R, and tea grape groups were exposed to 60-min shock, 60-min ischemia, and 120-min reperfusion^[16]^. Rats were anesthetized using 50 mg/kg intraperitoneal (i.p) ketamine hydrochloride (HCl) (Ketalar®, Eczacıbaşı Parke-Davis, Istanbul, Turkey) and 10 mg/kg i.p xylazine HCl (Alfazyne®, Alfasan International B.V. Woerden, the Netherlands) and were immobilized in a supine position under a heating lamp. The surgical area was shaved and sterilized with povidone-iodine solution. Median laparotomy was performed, after which the right carotid artery was cannulated with a 22G Intracath for arterial pressure monitoring. We also cannulated the left internal jugular vein for blood and fluid replacement. The arterial line was attached to a monitor with a transducer set, and pressure was monitored in an invasive manner until the end of the experiment. Following the catheterization procedure, the shock was established by collecting blood from the carotid artery into an injector containing 500 international units (IU) heparin until a value of mean arterial pressure (MAP) ≤ 50 mm Hg was achieved, thus replicating aneurysm rupture and hemorrhagic shock. The blood was stored at room temperature for subsequent use in resuscitation. The shock was maintained for 60 min once MAP ≤ 50 mmHg was attained. Blood was again collected as necessary during this procedure in order to ensure that the MAP was maintained at ≤ 50 mmHg, and the quantities and timings involved were recorded. The abdominal aorta was explored by means of median laparotomy toward the conclusion of the shock-induction stage. Anticoagulation was established (ischemia + resuscitation stage) with intravenous administration of 100 IU heparin to all animals at the end of exploration. At the end of the 60-min shock-induction stage, ischemia was induced in all subjects by placing bulldog clamps on the infrarenal abdominal aorta and the iliac bifurcation. Resuscitation commenced at the time of clamping and by returning half of the blood collected. The ischemic process in this model is intended to replicate surgical treatment, halting bleeding using an aortic clamp, and surgical reconstruction. The rats in the sham group received 1 ml glycerol i.p per day for five days before I/R. Rats in the tea grape group received tea grape 100 ml/kg/day i.p for five days before I/R^[15]^. Rats in the control group were anesthetized and fixed in a supine position under a heating lamp. A midline laparotomy was then performed. Blood was collected from all rats for biochemical analysis from the right ventricle by means of median sternotomy. All rats were sacrificed by exsanguination from the carotid artery at the conclusion of the experiment.

### Biochemical Investigations

#### Pulmonary MDA and GSH Level Measurement

Lung tissue specimens were washed in cold phosphate buffer and then homogenized for 1 min at 30 Hertz. Homogenized lung tissues were then centrifuged at 3000 g/15 min at +4 °C and the supernatant was removed. The Ellman’s method was used to measure pulmonary GSH levels^[17]^, while MDA was measured as described by Draper and Hadley^[18]^.

### Histopathological Analysis Procedure

Lung tissue specimens were fixed for 48 hours in 10% phosphate-buffered formalin solution (Sigma Aldrich, Germany), after which they were subjected to routine preparation procedures and embedded in hard paraffin blocks (Merck, Germany). A rotary microtome (Leica RM2525, Lecia, Germany) was then used to take 3-4 µm sections, which were then stained with Harris hematoxylin and eosin G (H&E; Merck, Darmstadt, Germany).

### Immunohistochemistry Analysis Procedure

Caspase-3 primary antibody (1:200, rabbit polyclonal, Caspase-3, Abcam, United Kingdom) and secondary antibody (Goat Anti-Rabbit IgG H&L-HRP, ab205718, Abcam, United Kingdom) kits were used to identify apoptotic pneumocytes.

Lung tissue sections were first subjected to deparaffinization, followed by antigen retrieval procedures as recommended by the manufacturer. They were then incubated for 60 min with primary and secondary antibodies. Lung tissue sections were finally stained with diaminobenzidine tetrahydrochloride (DAB, Sigma Chemical, St. Louis, Missouri, United States of America) and Harris hematoxylin (Merck, Germany).

### Semi-quantitative Analysis

Lung tissue damage scoring was performed on the basis of alveolar histopathological scores with a modification of the lung damage score of Matute-Bello et al.^[19]^ (Table 1). Twenty randomly selected areas on each slide were analyzed in a blinded fashion. In addition, Caspase-3 positivity of pneumocytes was scored as none (< 5%; 1), mild (5-25%; 2), moderate (26-50%; 3), and severe (50%; 4). At least five microscopic fields were examined for scoring. Thirty-five randomly selected areas on each slide were analyzed in a blinded fashion by two histopathologists.

**Table 1:** Modified (from Matute-Bello et al.[19]) Alveolar and Lung Histopathological Damage Score (ALHDS).

Findings	Score		
	0	1	2
Edema in interstitial areas	None (< 5%)	Mild (6-50%)	Severe > 50%
Vascular congestion	None (< 5%)	Mild (6-50%)	Severe > 50%
Alveolar septum thickness (treatment group/control group)	≤ x1	x1-x2	≥ x2
Inflammation	None (< 5%)	Mild (6-50%)	Severe > 50%

### Quantitative Analysis

Alveolar wall thickness (µm^2^) was calculated using the Olympus DP2-BSW (Ver.2.1 to Ver.2.2, Build 6212, Tokyo, Japan) software system by two blinded histologists.

### Statistical Analysis

Semi-quantitative and biochemical data were analyzed using IBM SPSS Statistics software (IBM, New York, United States of America), version 18.0. Non-parametric data elicited by the semi-quantitative analysis were calculated as a median (interquartile range), and the differences between groups were analyzed using the Kruskal-Wallis test followed by the Tamhane’s T2 test. *P* values ≤ 0.05 were regarded as significant.

## RESULTS

### Biochemical Results

**Fig. 1:**
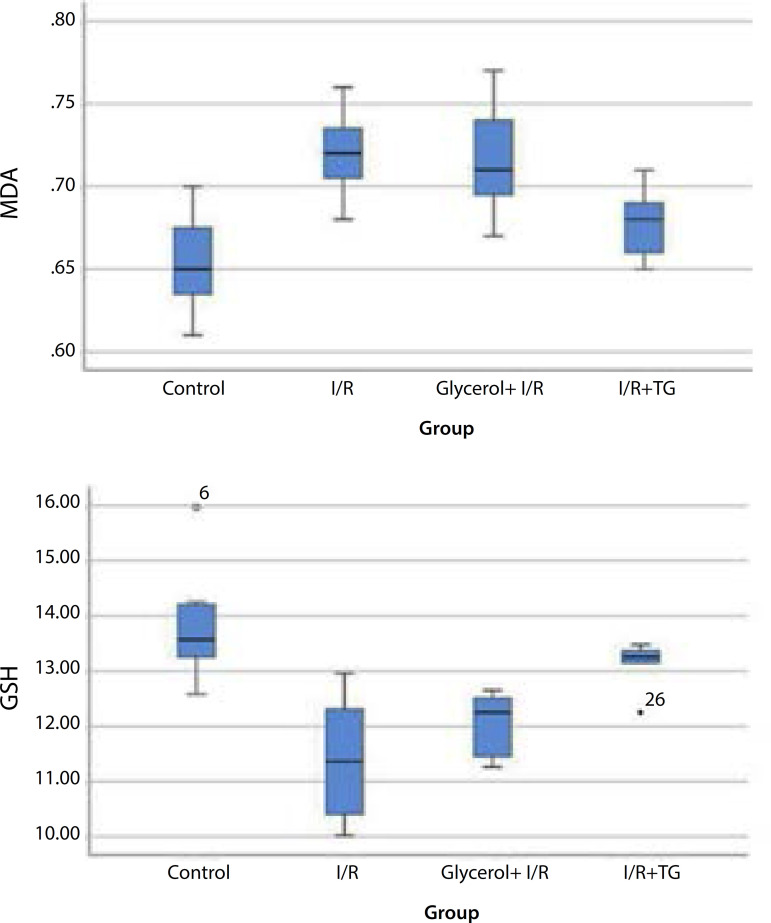
Graphs comparing the median and interquartile range of MDA and GSH between the groups.

Pulmonary tissue MDA levels increased from 0.65 (0.62-0.69) µmol/g tissue in the control group to 0.72 (0.70-0.74) µmol/g tissue in the I/R group and to 0.71 (0.69-0.75) µmol/g tissue in the glycerol + I/R group (Figure 1; *P*=0.01 and *P*=0.028, respectively). We observed no significant difference between the I/R and the glycerol + I/R group MDA levels (Figure 1; *P*=1.00). However, tea grape treatment reduced the MDA level from 0.72 (0.70-0.74) µmol/g tissue in the I/R group to 0.68 (0.65-0.70) µmol/g tissue (Figure 1; *P*=0.009).

GSH levels in pulmonary tissue decreased from 13.56 (13.26-14.26) µmol/g tissue in the control group to 11.37 (10.15-12.36) µmol/g tissue in the I/R group and to 12.25 (11.26-12.56) µmol/g tissue in the glycerol + I/R group (Figure 1; *P*=0.009 and *P*=0.019, respectively). There was no significant difference between GSH levels in the I/R and the glycerol + I/R groups (Figure 1; *P*=0.817). Tea grape treatment increased GSH levels from 11.37 (10.15-12.36) µmol/g tissue in the I/R group to 13.26 (13.15-13.48) µmol/g tissue (Figure 1; *P*=0.037).

### Light Microscopy Results

**Fig. 2:**
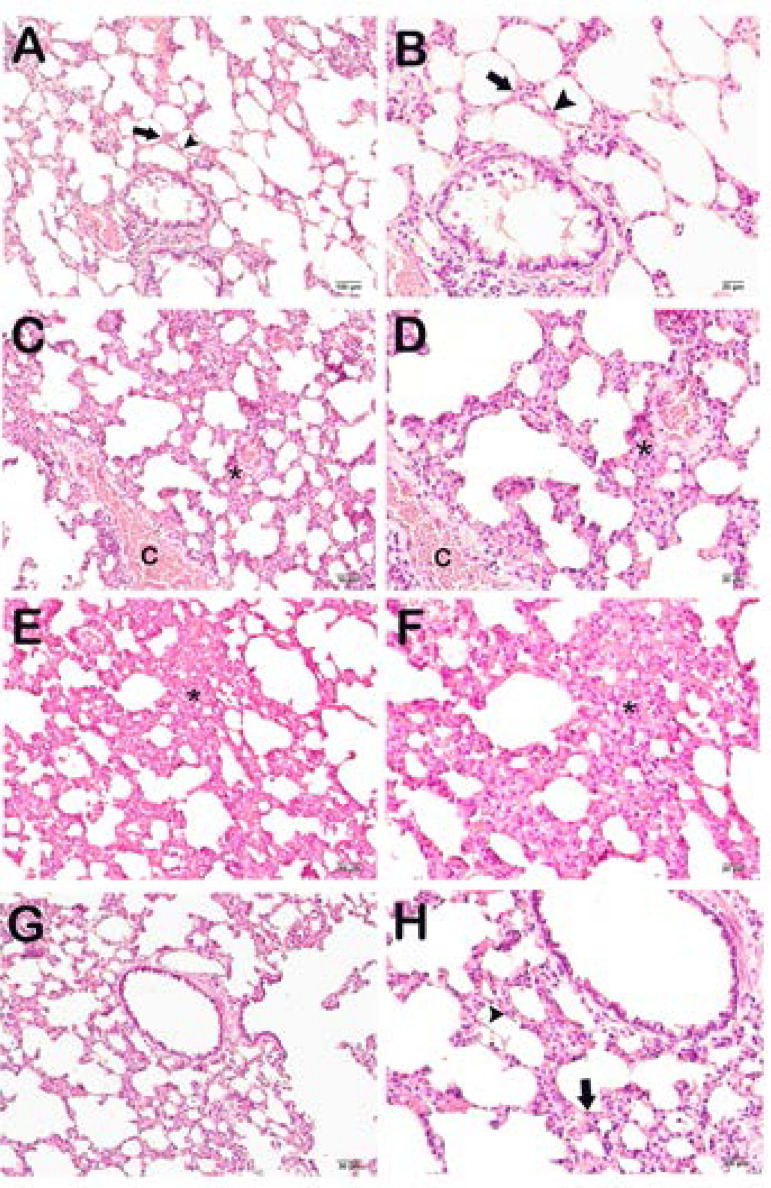
Representative light microscopic images of H&E-stained lung tissue sections. Type I pneumocytes (arrow), Type II pneumocytes (arrowhead). A(x20)-B(x40): Normal lung tissue histology in control group sections. [ALHDS score median: 1 (1-1.5)]. C(x20)-D(x40): Sections from the I/R group show diffuse edematous areas (asterisk) in interalveolar regions and vascular congestion (c). [ALHDS score median: 6.5 (5-7.5)]. E(x20)-F(x40): Sections from the I/R+Glycerol group exhibit thickening of the alveolar septal wall and edema interalveolar area (asterisk). Vascular congestion (c) can also be seen [ALHDS score: 6 (5-6.5)]. G(x20)-H(x40): Sections from the TG group exhibit decreased thickening [ALHDS score: 3 (2-3)] of the alveolar septal wall and edema.

Pulmonary tissue alveoli exhibited a normal appearance in the control group (Figure 2A and B); the mean alveolar septal thickness was 9.62 (7.56-10.10) µm and the median Alveolar and Lung Histopathological Damage Score (ALHDS) was 1 (1-1.5). Diffuse edematous areas in interalveolar regions and vascular congestion were observed in the I/R group (Figure 2C and D); median ALHDS score was 6.5 (5-7.5). We also observed thickening in the alveolar septal wall; mean alveolar septal thickness was 19.45 (18.04-21.82) µm. Similarly, we observed edema and vascular congestion in interstitial areas and alveolar septal wall thickening in the glycerol + I/R (sham) group (Figure 2E and F); mean alveolar septal thickness was 19.35 (18.63-22.58) µm and median ALHDS score was 6 (5-6.5). In contrast, the tea grape treatment group exhibited decreased alveolar septal wall thickness, together with reduced interstitial edematous areas and vascular congestion (Figure 2G and H); mean alveolar septal thickness was 10.10 (7.81-11.25) µm and median ALHDS score was 3 (2-3).

### Semi-quantitative Analysis

**Fig. 3:**
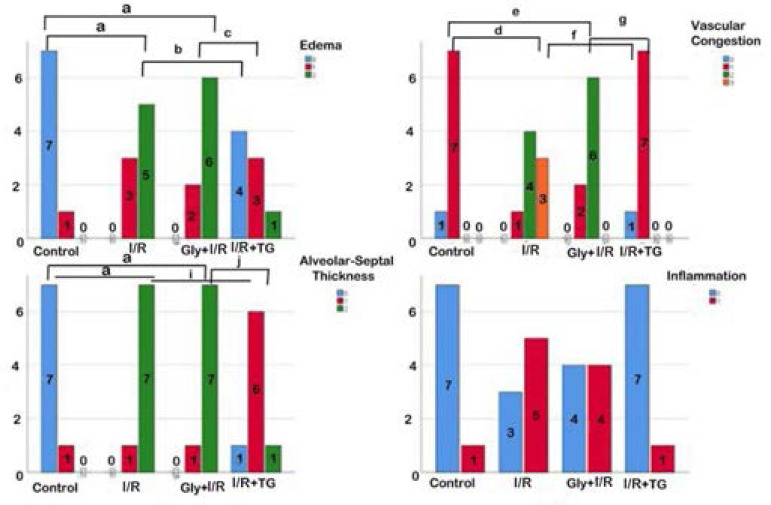
Comparison of the semi-quantitative analysis results between the groups. Median ± 25%-75% interquartile range. aP=0.00; compared with the control group. bP=0.05; compared with the ischemia/reperfusion (I/R) group. cP=0.021; compared with the glycerol (Gly) + I/R group. dP=0.003; compared with the control group. eP=0.006; compared with the control group. fP=0.003; compared with the I/R group. gP=0.006; compared with the Gly + I/R group. hP=0.002; compared with the I/R group. iP=0.013; compared with the I/R group. jP=0.0013; compared with the Gly + I/R group. kP=0.01; compared with the I/R group. lP=0.00; compared with the Gly + I/R group. Kruskal-Wallis - Tamhane’s T2 test. TG=tea grape

We observed an increase in edematous areas in the interalveolar region and vascular congestion in the I/R and the glycerol + I/R groups compared to the control group (Figures 2A to F and Figure 3; *P*<0.05). There was no difference between the I/R and the glycerol + I/R groups in terms of interstitial edema, vascular congestion, or ALHDS scores (Figure 2C to F and Figure 3; *P*>0.05). In the tea grape group, there was a decrease in ALHDS scores in association with increased edematous areas in the interalveolar region and vascular congestion (Figure 2C to H and Figure 3; *P*<0.05).

### Quantitative Analysis

**Fig. 4:**
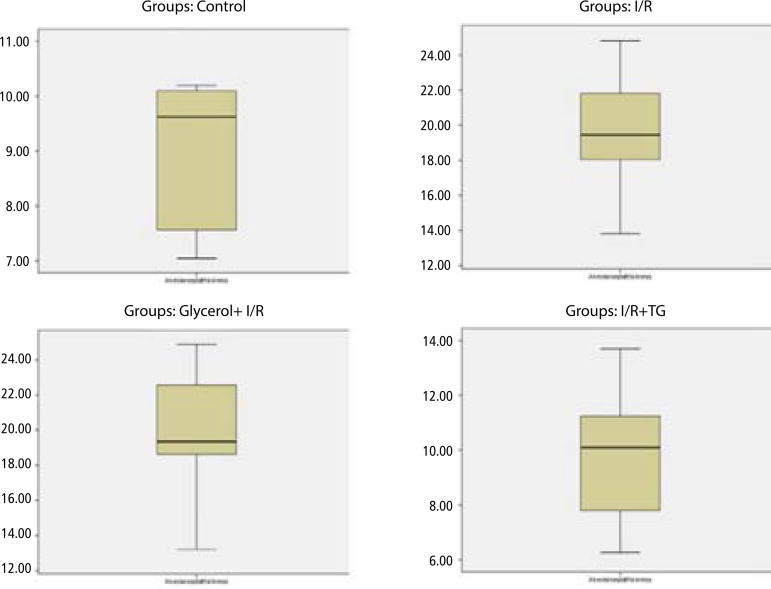
Comparison of the median alveolar septum thickness between the groups.

Measurements involving pulmonary tissue sections revealed that alveolar wall thickness increased from 9.62 (7.56-10.10) µm in the control group to 19.45 (18.04-21.82) µm in the I/R group and to 19.35 (18.63-22.58) µm in the glycerol + I/R group (Table 2 and Figure 4; *P*=0.000 and *P*=0.000, respectively). There was no difference in septal wall thicknesses between the I/R and the glycerol + I/R groups (Table 2 and Figure 4; *P*=0.989). Alveolar septal wall thickness decreased from 19.45 (18.04-21.82) µm in the I/R group to 10.10 (7.81-11.25) µm in the tea grape treatment group (Table 2 and Figure 4; *P*=0.000).

**Table 2:** Alveolar septum thickness analysis results.

Group	Alveolar wall thickness (µm) (median and interquartile range)	Alveolar septum thickness (treatment group/control group)	Matute-Bello et al.^[19]^ modified Alveolar Septum Thickness Score
Control	9.62 (7.56-10.10)	1.00	0 (<x2)
I/R	19.45 (18.04-21.82)^a^^,^^d^	2.02	2 (≥ x2)
Glycerol + I/R	19.35 (18.63-22.58)^a^	2.01	2 (> x2)
I/R + tea grape	10.10 (7.81-11.25)^b^^,^^c^	1.05	1 (x1-x2)

a *P*=0.000; compared with the control group.

b *P*=0.000; compared with the ischemia/reperfusion (I/R) group.

c *P*=0.000; compared with the glycerol + I/R group.

d *P*=0.989; compared with the glycerol + I/R group.

Kruskal-Wallis - Tamhane's T2 test.

### IHC Results

**Fig. 5:**
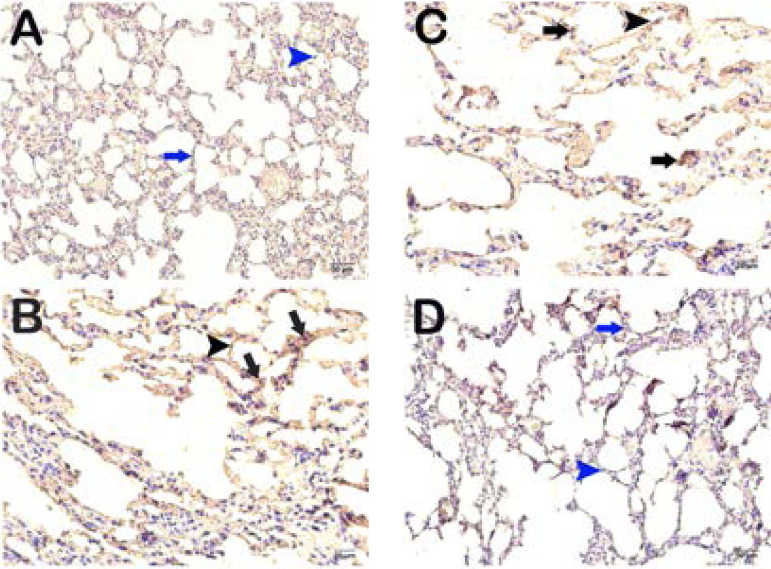
Representative light microscopic images of caspase-3 antibody-stained lung tissue sections. A: Sections from the control group exhibit normal Type I (blue arrowhead) and Type II pneumocytes (blue arrow) [median caspase-3 positivity score: 0.00 (0-1)]. x40. B: Sections from the I/R group showing intense caspase-3 positivity in Type I (black arrowhead) and Type II pneumocytes (black arrow) [median caspase-3 positivity score: 2.00 (2-2.5)]. x40. C: Glycerol+I/R group sections showing intense apoptosis in Type I (black arrowhead) and Type II pneumocytes (black arrow) [median caspase-3 positivity score: 2.00 (2-2)]. x40. D: Sections from the TG treatment group showing decrease caspase-3 positivity in Type I (blue arrowhead) and Type II pneumocytes (blue arrow) [median caspase-3 positivity score: 1.00 (0-1)]. x40.

**Fig. 6:**
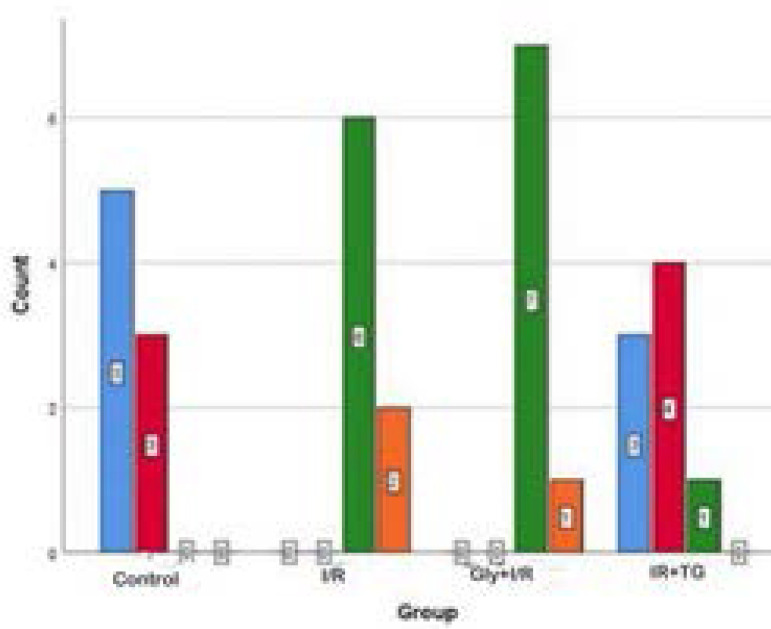
Distribution of the median grade scores of caspase-3 positivity between groups. Gly=glycerol; I/R=ischemia/reperfusion; TG=tea grape

Type I and type II pneumocytes in pulmonary tissue sections from the control group exhibited a normal structure (Figure 5A and Figure 6); median caspase-3 positivity score was 0.00 (0-1). The I/R group showed an increase in apoptotic type I and type II pneumocytes compared to the control group (Figure 5B and Figure 6); median caspase-3 positivity score was 2.00 (2-2.5) (*P*=0.00). Similarly, there was an increase in apoptotic type I and type II pneumocytes showing caspase-3 activity in the glycerol + IR compared to the control group (Figure 5C and Figure 6); median caspase-3 positivity score was 2.00 (2-2) (*P*=0.00). There was no difference between apoptotic pneumocytes in terms of caspase-3 positivity between the I/R and glycerol + I/R groups (Figure 5C and Figure 6) (*P*=0.992). However, tea grape treatment resulted in a significant decrease in type I and type II pneumocytes in terms of caspase-3 positivity compared to the I/R group (Figure 5D and Figure 6); median caspase-3 positivity score was 1.00 (0-1) (*P*=0.03).

## DISCUSSION

Previous studies reported alveolar dilatation in lung tissue, vascular congestion, lymphocyte infiltration, hyaline cast structure, alveolar wall thickening, and degenerating epithelial cells as a result of aortic occlusion^[20,21]^. Erer et al.^[22]^ reported pulmonary alveolar debris accumulation and inflammation and edematous areas in interstitial regions following aortic occlusion. Similarly, in their study of aortic cross-clamp-induced lung injury, Kurt et al.^[21]^ observed vascular congestion, edema, and inflammation in interstitial areas. However, they reported no alveolar hyaline cast structure or alveolar wall thickening, although alveolar dilations were present. In contrast, Findik et al.^[23]^ reported that aortic occlusion caused infiltration of inflammatory cells and alveolar wall thickening. Similarly, in our study, we observed significant edema in interstitial areas, vascular congestion, and alveolar wall thickening, but no alveolar dilation or inflammation. We think that since our study concluded shortly after I/R application, there was insufficient time (at least 6-72 hours) for inflammatory changes to develop.

Although the mechanism involved in pulmonary tissue damage following aortic occlusion is not fully understood, studies have shown that oxidative stress, in which an increase in ROS production and a decrease in antioxidant enzyme activation occur, may be the cause^[11,24]^. Kurtoglu et al.^[25]^ determined that aortic occlusion resulted in an increase in myeloperoxidase and MDA levels in serum and lung tissue. Similarly, we observed that aortic occlusion increased MDA levels in pulmonary tissue. No studies have investigated GSH levels in aortic occlusion-related lung damage, although GSH levels have been reported to decrease following reperfusion^[8]^. In contrast, no significant difference in GSH levels have been reported in a clinical study examining abdominal aortic aneurysm repairs^[26]^. In the present study, we observed a decrease in pulmonary GSH levels following aortic occlusion.

Previous studies have reported that oxidative stress formation in association with ROS production as a result of I/R leads to apoptosis. Findik et al.^[23]^ demonstrated that abdominal aorta I/R resulted in apoptosis in pneumocytes in lung tissue. Kurt et al.^[21]^ observed that aortic cross-clamping resulted in apoptosis in pulmonary cells by increasing caspase-3 expression. In our study, we determined an increase in caspase-3 positivity in type I and type II pneumocytes following aortic occlusion.

In addition to antioxidant properties, the tea grape, a member of the bilberry (*V. myrtillus*) family, has also been reported to exhibit anti-inflammatory and anticarcinogenic characteristics^[15,27]^. There are no reports on the effect of tea grape on lung damage resulting from aortic occlusion. In addition, only a few studies have investigated I/R-induced small intestine and ovary injury^[28]^. In one of these studies, Jakesevic et al.^[29]^ showed that bilberry prevented I/R-induced small intestine injury by inhibiting LPO. In their study of restraint stress-induced liver damage, Bao et al.^[30]^ reported that bilberry extract exhibited a protective effect against oxidative stress by reducing mitochondrial membrane potential-associated sodium/potassium adenosine triphosphatase activity, and mitochondrial complex II activity that increased in association with ROS production. They also reported that bilberry lowered nitric oxide levels^[30]^. In another study, Jakesevic et al.^[31]^ reported that it exhibited a powerful protective effect against I/R-induced small intestine injury resulting from oxidative stress and inflammation induced in the small intestine via I/R. Similarly, our study showed that it reduced oxidative stress by lowering lung tissue MDA levels that increased as a result of aortic occlusion. We also observed that tea grape increased pulmonary GSH levels. While no previous study investigated the protective effects of tea grape against apoptosis in pneumocytes in lung tissue resulting from aortic occlusion, Kara et al.^[28]^, in their study of ovarian I/R, reported that tea grape reduced the numbers of apoptotic follicle cells induced by I/R. Wang et al.^[32]^ reported that bilberry reduced caspase-3 expression. Similarly, Ogawa et al.^[13]^ suggested that bilberry inhibited ROS production and the activation of pro-apoptotic proteins such as caspase-3/7. Similarly, in our study, tea grape reduced the caspase-3 positivity in pneumocytes that increased in association with aortic occlusion.

This study is the first to investigate the effects of tea grape in I/R-induced lung injury. We also examined oxidative stress and apoptotic damage pathways using biochemical, histopathological, IHC, and quantitative measurement methods.

### Limitations

The limitations of our study include use of glycerol, that is tea grape solvent, as sham. Larger groups may need to be used to study our findings with additional oxidant and antioxidant molecules and enzymes, proinflammatory cytokines, and mitochondrial Ca^+2^ levels.

## CONCLUSION

Our findings may result in a new perspective regarding the oxidative stress developing in association with LPO and apoptosis associated with caspase-3 expression induced in lung injury by I/R. Tea grape protected against the lung injury resulting from aortic occlusion-induced I/R by reducing LPO and apoptosis.

**Table:** 

Authors' roles & responsibilities	
DH	Substantial contributions to the conception or design of the work; or the acquisition, analysis, or interpretation of data for the work; drafting the work or revising it critically for important intellectual content; final approval of the version to be published
SE	Substantial contributions to the conception or design of the work; or the acquisition, analysis, or interpretation of data for the work; final approval of the version to be published
SOK	Substantial contributions to the conception or design of the work; or the acquisition, analysis, or interpretation of data for the work; final approval of the version to be published
TM	Substantial contributions to the conception or design of the work; or the acquisition, analysis, or interpretation of data for the work; drafting the work or revising it critically for important intellectual content; final approval of the version to be published
LT	Substantial contributions to the conception or design of the work; or the analysis, or interpretation of data for the work; final approval of the version to be published
AY	Substantial contributions to the conception or design of the work; or the analysis, or interpretation of data for the work; final approval of the version to be published
KA	Substantial contributions to the conception or design of the work; or the analysis, or interpretation of data for the work; final approval of the version to be published
